# Black and White Women in Maryland Receive Different Treatment for Cervical Cancer

**DOI:** 10.1371/journal.pone.0104344

**Published:** 2014-08-14

**Authors:** Saroj Fleming, Nicholas H. Schluterman, J. Katthleen Tracy, Sarah M. Temkin

**Affiliations:** 1 University of Maryland School of Medicine, Department of Obstetrics, Gynecology and Reproductive Sciences, Baltimore, Maryland, United States of America; 2 University of Maryland School of Medicine, Department of Epidemiology and Public Health, Baltimore, Maryland, United States of America; Old Dominion University, United States of America

## Abstract

**Purpose:**

Despite an overall decrease in incidence, the death rate from cervical cancer in the United States remains higher in black women than their white counterparts. We examined the Maryland Cancer Registry (MCR) to determine treatment factors that may explain differences in outcomes between races in the state of Maryland.

**Methods:**

Incident cervical cancers in the MCR 1992–2008 were examined. Demographics, tumor characteristics and treatments were compared between races and over time.

**Results:**

Our analysis included 2034 (1301 white, 733 black) patients. Black women were more likely to have locally advanced or metastatic disease at diagnosis (p<0.01). They were more likely to receive any radiation or chemotherapy combined with radiation and less likely to receive surgery (p<0.01). When adjusted for stage and insurance status black women had 1.50 (95% CI 1.20–1.87) times the odds of receiving radiation and 1.43 (95% CI 1.11–1.82) times the odds of receiving chemotherapy. Black women with cervical cancer had 0.51 times the adjusted odds (95% CI 0.41–0.65) of receiving surgery compared to white women. Racial differences in treatment did not change significantly over time.

**Conclusions:**

Surgical treatment for newly diagnosed cervical cancer in the state of Maryland was significantly less common amongst black women than white during our study period. Equivalent treatments are not being administered to white and black patients with cervical cancer in Maryland. Differences in care may contribute to racial disparities in outcomes for women with cervical cancer.

## Introduction

In 2014, an estimated 12,360 women will be diagnosed with cervical cancer in the United States. Despite a decreased overall incidence for the last 50 years, a disparity in incidence persists between white and black women (7.8 versus 10.4 per 100,000, age-adjusted to the 2000 US standard population). In addition to differences in incidence rates, mortality from cervical cancer remains nearly twice as high in black women than in their white counterparts (4.3 versus 2.2 per 100,000) in the United States [1]–[3].

Several factors have been postulated as the cause of these racial disparities. One explanation for the differences in incidence has been differences in rates of screening [4]. Recent data, however, demonstrates that pap smear screening rates have become quite similar between white and black women [5]–[7]. Differences in mortality have been often been ascribed to variation in stage at diagnosis. Black women are more likely to be diagnosed with regional or distant disease and less likely to be diagnosed with local disease [1], [2], [4]. Treatment differences have also been documented as playing a role in cervical cancer disparities. Black women are less likely to receive a radical hysterectomy for early staged disease and less likely to complete brachytherapy than white women [8], [9]. Several authors have suggested, however, that when social factors are accounted for (e.g. socioeconomic factors and medical comorbidities) cervical cancer mortality is quite similar between racial groups [10], [11].

Maryland, similar to the United States as a whole, has seen an overall decreased incidence in cervical cancer in both white and black patients for decades. However, cervical cancer mortality increased between 2003 and 2007 amongst black women (at a rate of 2.8% per year) whereas it decreased amongst white women at a rate of 0.1% per year [12]. The State of Maryland supports cervical cancer screening services to uninsured or underinsured low-income women residents. Cervical cancer screening in Maryland is provided to the majority of women, with 90.4% reporting Pap smear screening within the last 3 years [12]. These self-reported rates are the same in white and black patients. In addition, State funding is designated to provide breast and cervical cancer treatment for low-income uninsured or underinsured women living in Maryland.

We undertook this study to determine whether specific treatment differences between black and white patients in the state of Maryland can be identified to explain racial disparities in cervical cancer mortality. We additionally examined whether these differences have changed over time.

## Materials and Methods

Data were obtained from the Maryland Cancer Registry (MCR). The Maryland Cancer Registry is a computer-based cancer incidence data system maintained under the direction of the Maryland Department of Health and Mental Hygiene. The data system originated in 1982, with mandatory reporting starting in 1991. Maryland law now requires hospitals, freestanding radiation therapy centers, ambulatory care facilities, laboratories and physicians to report tumors to the MCR within 6 months of diagnosis [3], [13].

This retrospective study was approved by the Institutional Review Board at the University of Maryland Baltimore (HP-00048096). As the data obtained from the MCR was anonymized and de-identified prior to analysis, consent from patients was not obtained.

A dataset containing a de-identified list of all incident cervical cancer tumors during the time period from 1992–2008 was compiled from the MCR. Demographic information included the following: age at diagnosis, insurance status, census tract income, and medical comorbidities. Data regarding race/ethnicity (black, white or other); summary staging (localized, regional or distant); insurance status (private, public or uninsured); histology (squamous, non-squamous) were also obtained. Tumor stage was classified according to the 2000 SEER Summary Staging Manual [14]: Summary Stage 1 was classified as local, Summary Stages 2–5 were classified as Regional, and Summary Stage 7 was classified as Distant. Treatment data were collected as well, and included the following: surgery, external beam radiation, brachytherapy, and chemotherapy.

Cervical cancer incidence was calculated by race for each year from 1992–2008 and for the overall study period, and was expressed in terms of annual number of cases per 100,000 women age 20 years and older. US Census data for the state of Maryland was used to provide the denominators [15]. Because yearly population estimates were not available during the earlier years of the study time period, the race-specific state population for those years was estimated using a linear trend from the 1990 to 2000 published figures.

The majority of data regarding insurance and treatment variables were missing in the years 1992–1998, and not enough patients of races other than white or black were present to conduct meaningful comparisons between races. Therefore, the remainder of the study focused on data from black or white patients diagnosed in the years from 1999–2008. Races were compared by socio-demographic variables and tumor characteristics using chi-square tests. Where noted in the text, additional chi-square tests assessed whether the two races were statistically different along a single category of a multi-level variable.

The proportions of patients receiving different treatment combinations were compared between different race and tumor stage categories. To test for trends in treatment disparities over time, the sample was divided into two five-year periods: 1999–2003, and 2004–08. Differences in treatment receipt within a race between time periods were assessed using chi-square tests. A series of Breslow-Day tests then revealed whether a race-time interaction affected treatment receipt (i.e. whether treatment disparities between races were different between time periods). Adjusted analysis using logistic regression accounted for the effects of confounding variables, yielding odds ratios of the independent effects of race on receipt of a specific treatment. Potential confounders were chosen for the regression model via backward selection, with a likelihood ratio p-value of <0.05 considered sufficient for inclusion into the model.

In the analysis comparing treatments between races, the primary data set included 2034 black or white patients diagnosed from 1999–2008. Those missing data for tumor stage, tumor grade, or health insurance status were considered a separate category for each of these variables. When comparing treatments between races in the unadjusted analyses, the analysis was performed first without those patients missing data for the relevant tumor or insurance variables, and then again with those patients as a separate “missing” category. This latter approach allowed us to assess the effect of missing data on the results. When no meaningful differences were found between the analyses with and without the missing data, only those analyses that excluded the missing data were presented. In contrast to the unadjusted analyses, the logistic regression models always included those who were missing tumor or insurance data as a separate category, so that results would be more stable within each model and more directly comparable between models. However, because the regression models used treatment as the outcome, each model excluded those who were missing the relevant treatment data.

All analyses were conducted with SAS version 9.2 (Cary, NC, USA). For all statistical tests, a p-value of <0.05 was considered statistically significant.

## Results

**Figure 1:**
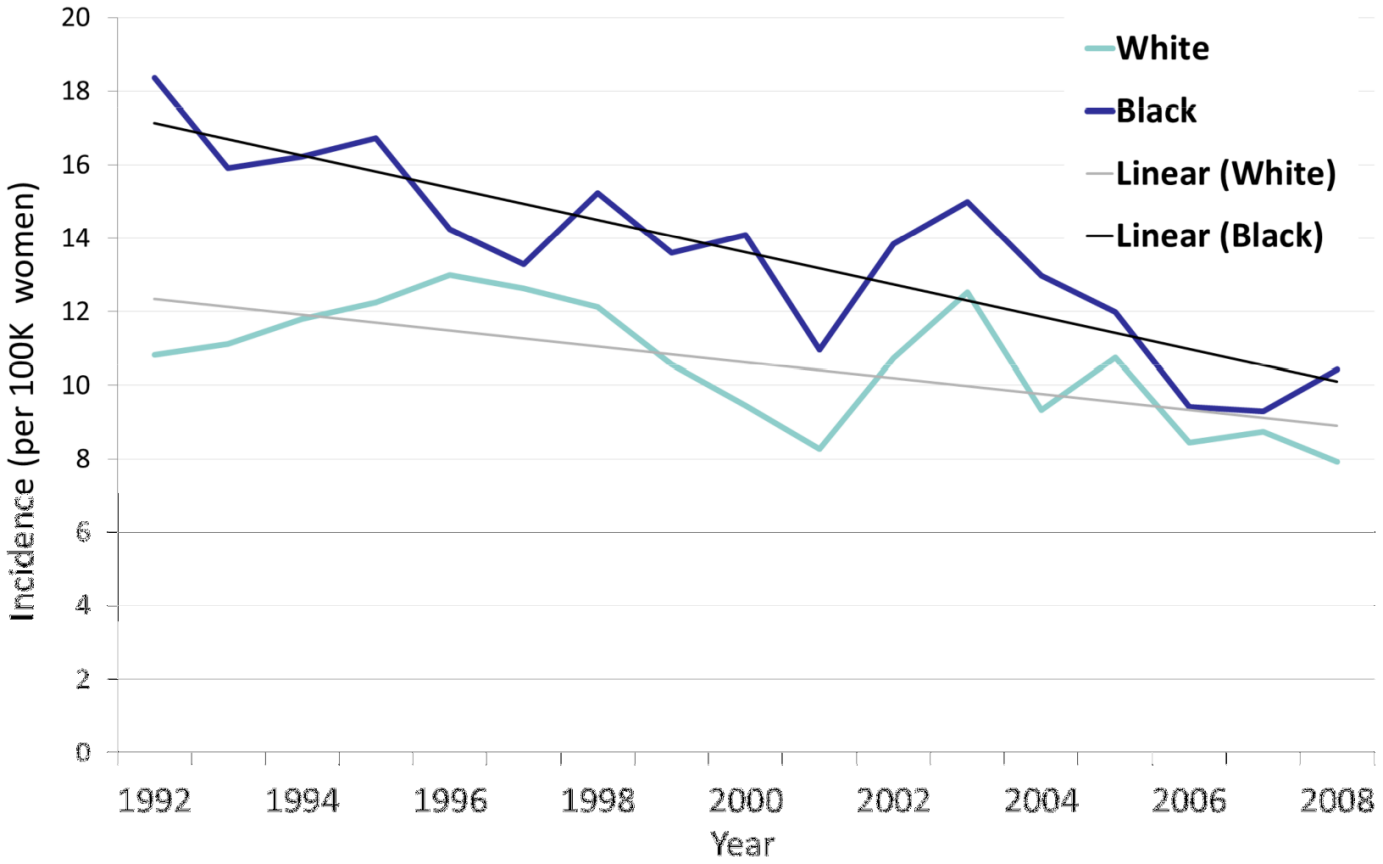
Annual incidence of cervical cancer in Maryland by race for women 20 and older, 1992–2008.

Four thousand forty-eight cases of cervical cancer were diagnosed in Maryland between 1992 and 2008. The average annual incidence of cervical cancer in the state of Maryland from 1992–2008 was 10.6 cases per 100,000 women for whites, and 13.3 cases per 100,000 for blacks (Figure 1). The incidence of cervical cancer declined during this time period amongst both white (n = 2432) and black (n = 1318) patients.

The subsequent analysis, which assessed predictors of treatment, included 2034 incident cancers diagnosed from 1999 to 2008 amongst white or black patients. Of these patients, 407 (20%) were missing data for health insurance type, 794 (39%) were missing data for tumor grade, and 432 (21%) were missing data for tumor stage. Table 1 provides the characteristics of these patients. Age was similar between races. The ratio of white to black patients diagnosed with cervical cancer by two year period remained relatively constant during this ten year period (p = 0.91). Black patients were more likely to be publically insured (p<0.01) and diagnosed with squamous cell carcinoma as opposed to adenocarcinoma (p<0.01). Black patients were less likely to be diagnosed with local disease (p<0.01) than white patients.

**Table 1:** Patient characteristics between 1999–2008.

Characteristic		White	Black	p-value*
**Number**		1301	733	
**Age, mean (SD)**		51.9 (16.5)	53.1 (15.7)	0.15^t^
**Diagnosis Year, row % (n)**	1999–2000	63.0 (269)	37.0 (158)	0.91
	2001–2002	63.6 (255)	36.4 (146)	
	2003–2004	63.5 (294)	36.5 (169)	
	2005–2006	66.1 (259)	33.9 (133)	
	2007–2008	63.8 (224)	36.2 (127)	
**Insurance Type, column % (n)**	Public	30.9 (319)	39.8 (236)	**<0.01**
	Private	38.0 (393)	32.4 (192)	
	Insured, type unknown	25.7 (266)	19.9 (118)	
	None	5.4 (56)	7.9 (47)	
	Missing	20.5 (267)	19.1 (140)	
**Histology type (ICD-O-3), column % (n)**	Squamous cell	58.7 (764)	71.1 (521)	**<0.01**
	Adenocarcinoma	23.5 (306)	14.1 (103)	
	Other/NOS	17.8 (231)	14.9 (109)	
**Grade, column % (n)**	I	15.8 (122)	9.9 (46)	**0.01**
	II	38.8 (300)	41.9 (195)	
	III	45.5 (352)	48.3 (225)	
	Missing	40.5 (527)	36.4 (267)	
**Stage, column % (n)**	Local	54.3 (538)	46.5 (284)	**<0.01**
	Regional	33.8 (335)	39.3 (240)	
	Distant	11.9 (118)	14.2 (87)	
	Missing	23.8 (310)	16.6 (122)	

*Chi-square test, except where noted, excluding those missing data.

t Student’s t-test.

Column percentages exclude those with missing data. Non-missing categories add up to 100%, except due to rounding.

**Figure 2:**
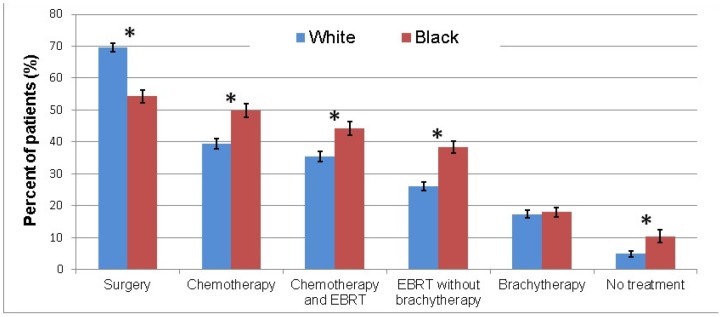
Differences in treatments received between races. White patients were more likely to receive surgery. Black patients were more likely to receive chemotherapy, chemotherapy and radiation, and radiation without brachytherapy. Categories are not mutually-exclusive nor exhaustive. Error bars represent standard error of population proportion. EBRT: external beam radiation therapy. *Statistically significant difference between races (chi-square test p-value<0.05).

As shown in Figure 2, white patients were more likely to receive surgery as treatment for incident cervical cancer than black women (p<0.01). Black women were more likely to receive chemotherapy (p<0.01), chemotherapy with external beam radiation therapy (p<0.01), and radiation without brachytherapy (p<0.01) than white patients. Brachytherapy administration overall was similar between races. Black patients were more likely than white patients to receive no treatment (p<0.01).

**Figure 3:**
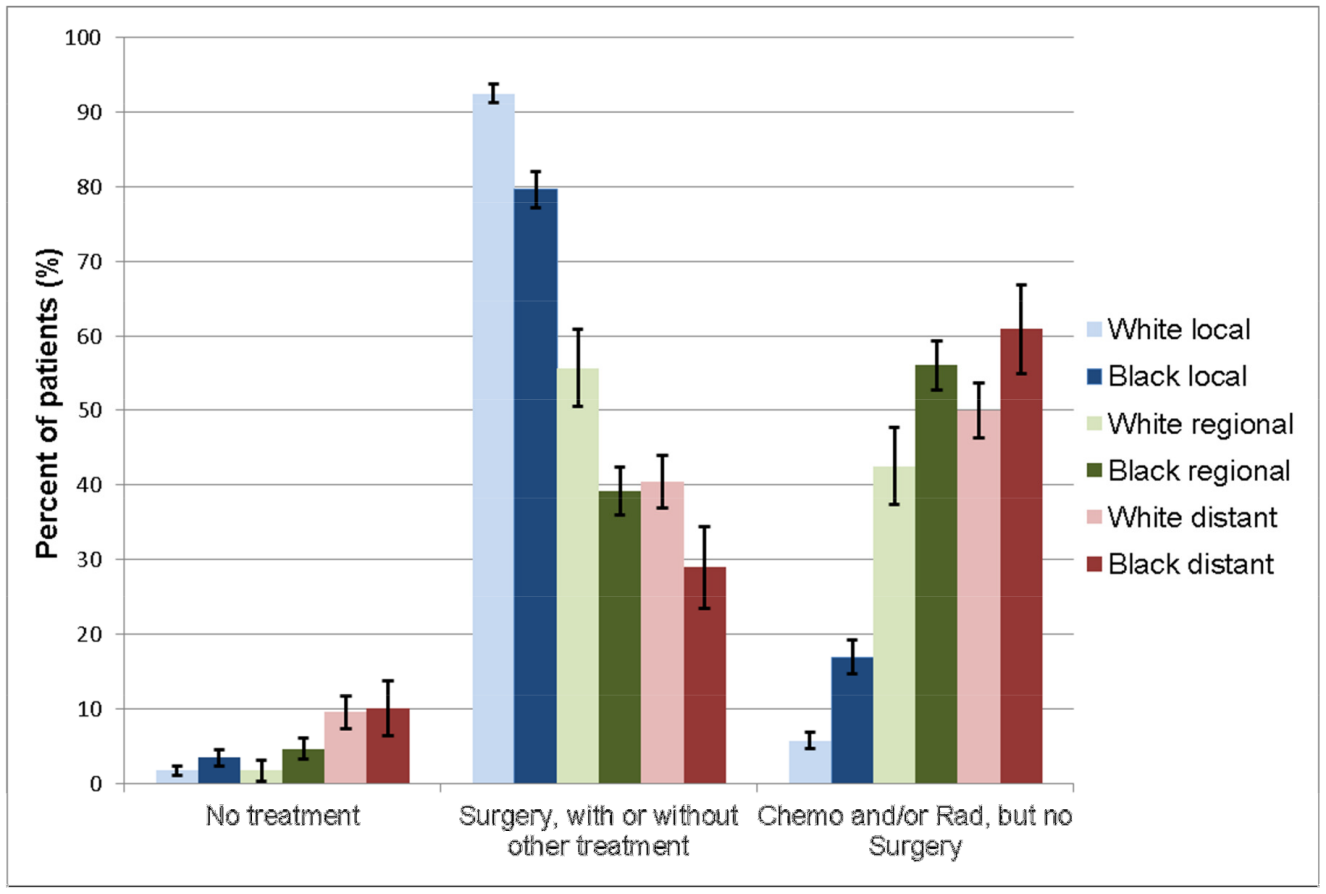
Differences in treatments received were still observed when stratified by extent of disease at presentation. Chemo: chemotherapy. Rad: radiation. Error bars represent standard errors of sample proportions.

These differences in the characteristics of treatment between white and black patients persisted when adjusted for extent of disease and insurance status (Figure 2). Regardless of stage at presentation, black women were more likely than white women to receive radiation or chemotherapy as their only treatment modality (Figure 3). White women were far more likely to receive surgery than black women, even amongst patients with local disease. White women were also more likely to receive multimodality treatment with surgery, chemotherapy and radiation than black women, regardless of extent of disease at presentation.

A series of logistic regression models were constructed using variables for race, tumor stage, and insurance status. Variables for histology and age were considered for inclusion in the model, but did not meet the significance threshold and were therefore dropped. When adjusted for stage and insurance status, black patients received different treatment than whites (Table 2). Black women had a substantially lower odds of receiving surgery (OR = 0.51 (95% CI 0.41–0.65)) and a higher odds of receiving chemotherapy (OR = 1.43 (95% CI = 1.11–1.82)), or external beam radiation (OR = 1.45 (95% CI = 1.16–1.87)). Results were similar whether missing variables were Included or excluded from the analysis.

**Table 2:** Adjusted^a^ odds ratios for receiving a particular treatment, among white or black patients, 1999–2008.

Characteristic		N	Chemotherapy	Any Radiation	EBRT	Brachytherapy	Surgery
**Total**		2034	43.2% (665/1536)	48.1% (877/1823)	30.5% (556/1823)	17.6% (321/1823)	64.0% (1130/1767)
**Race, OR (95% CI)**	White	1301	Ref.	Ref.	Ref.	Ref.	Ref.
	Black	733	1.43 (1.11–1.82)*	1.50 (1.20–1.87)*	1.45 (1.16–1.81)*	0.92 (0.71–1.20)	0.51 (0.41–0.65)*
**Stage, OR (95% CI)**	Local	822	Ref.	Ref.	Ref.	Ref.	Ref.
	Regional	575	11.44 (8.63–15.16)*	10.99 (8.40–14.37)*	10.73 (8.25–13.97)*	3.18 (2.39–4.23)*	0.15 (0.11–0.20)*
	Distant	205	10.76 (7.30–15.94)*	3.74 (2.68–5.21)*	4.16 (2.98–5.81)*	1.09 (0.69–1.74)	0.09 (0.06–0.13)*
	Missing	432	1.81 (1.24–2.65)*	1.27 (0.92–1.75)	1.33 (0.96–1.86)	0.81 (0.50–1.31)	0.10 (0.07–0.14)*
**Insurance, OR (95% CI)**	Public	555	Ref.	Ref.	Ref.	Ref.	Ref.
	Private	585	1.18 (0.87–1.59)	0.76 (0.58–1.00)	0.75 (0.57–0.99)	1.00 (0.74–1.36)	2.46 (1.86–3.26)
	Insured, type missing	384	0.94 (0.67–1.32)	0.52 (0.38–0.71)*	0.53 (0.39–0.72)*	0.74 (0.51–1.07)	2.31 (1.66–3.21)
	None	103	2.03 (1.17–3.53)*	1.08 (0.65–1.78)	0.97 (0.59–1.59)	1.10 (0.63–1.92)	0.60 (0.36–1.00)
	Missing	407	1.18 (0.75–1.83)	0.50 (0.34–0.72)*	0.46 (0.32–0.68)*	0.65 (0.40–1.05)	0.82 (0.55–1.22)

EBRT: external beam radiation therapy.

a Logistic regression models included variables for race, stage, and insurance type. A separate regression model was built for each treatment. Those missing data for stage or insurance type were included as a separate category. Those missing particular treatment data were excluded from the relevant regression models.

OR (95% CI): odds ratio (95% confidence interval).

*Odds ratio shows statistically significant effect (p<0.05 via logistic regression).

Treatment differences over time were noted in both races when the time period from 1999–2003 was compared to 2004–2008 (Table 3). All patients were more likely to receive chemotherapy in the second time period than the first. The percentage of white patients receiving radiation increased in 2004–2008 compared to 1999–2003. However, racial differences in treatment did not change significantly over time.

**Table 3:** Unadjusted treatment differences between 1999–2003 and 2004–2008.

		White			Black			
		1999–2003	2004–2008	p*	1999–2003	2004–2008	p*	p interaction**
**N**		693	608		394	339		
**Chemo, column % (n)**	Yes	32.2 (162)	47.3 (220)	**<0.01**	41.4 (116)	58.0 (167)	**<0.01**	0.88
	No	67.8 (341)	52.7 (245)		58.6 (164)	42.0 (121)		
	Missing	27.4 (190)	23.5 (143)		28.9 (114)	15.0 (51)		
**RT, column % (n)**	EBRT	23.2 (139)	29.1 (164)	**0.04**	38.9 (132)	37.7 (121)	0.34	0.45
	Brachy	17.0 (102)	17.8 (100)		15.9 (54)	20.3 (65)		
	No RT	59.8 (359)	53.1 (299)		45.1 (153)	42.1 (135)		
	Missing	13.4 (93)	7.4 (45)		14.0 (55)	5.3 (18)		
**Surgery, column % (n)**	Yes	69.2 (400)	70.0 (376)	0.77	56.5 (192)	51.9 (162)	0.24	0.28
	No	30.8 (178)	30.0 (161)		43.5 (148)	48.1 (150)		
	Missing	16.6 (115)	11.7 (71)		13.7 (54)	8.0 (27)		

RT: radiation therapy; EBRT: external beam radiation therapy; Brachy: brachytherapy.

*P - value excludes those missing data for particular variable.

**Breslow-Day test for interaction between race and time period regarding the effect on treatment receipt, excluding those missing data for particular variable.

Column percentages exclude those with missing data. Non-missing categories add up to 100%, except due to rounding.

### Analysis of missing data

The dataset included a large number of missing values for tumor characteristics (Table 1) and for treatment outcomes (Table 3). A series of analyses of missing data, reprising the analyses represented in Tables 1 and 3, were conducted that included those with missing data for these variables as a separate group, rather than exclude them altogether. In all of these analyses, the inclusion of a “missing” group did not meaningfully affect the results. Because no meaningful statistical differences were found during this additional analysis, only results excluding those missing data are presented here for any particular variable. Among the analyses presented here, only the logistic regression models represented in Table 2 analyzed as separate categories those who were missing data for tumor and insurance variables, but these models did not include those who were missing necessary data for the relevant treatment variables.

## Discussion

Our study examined the racial differences in cervical cancer treatment received in Maryland between 1999 and 2008. The overall incidence of cervical cancer decreased during this time period among black and white women. Black women presented with more advanced stage disease and were more likely to receive radiation than white women, but were less likely to receive brachytherapy. Unadjusted and adjusted for stage at presentation, however, white women were much more likely to receive surgical treatment for cervical cancer than black women. Our study suggests that equivalent treatments are not being administered to white and black patients with cervical cancer in Maryland. These treatment differences are likely to contribute the increased mortality seen in Maryland among black women with cervical cancer when compared to white women.

Several hypotheses have been proposed to account for poorer outcomes amongst black women with cervical cancer. Disparities in outcomes may result from unequal access to care and/or differences in the quality of care received. They may also result from variations in comorbidities that accompany a cancer diagnosis. Or, these differences in outcome may result from biologic distinctions between women of differing racial and ethnic backgrounds. Factors that ultimately contribute to differences in outcome include exposure to the HPV virus (which is causative of cervical cancer), access to high quality regular screening (the Pap test), and access to timely treatment – all factors which may be affected by race as well as socioeconomic differences.

Although lower educational attainment, older age, obesity, smoking, and neighborhood poverty have been found to be independently related to a decreased likelihood of recent pap screening [16], [17], black women in Maryland have similar rates of screening with pap smears as white women. Data from the Behavioral Risk Factor Screening System in Maryland show that since 2000, 84–90% of women adhere to Pap smear screening guidelines [12]. Infrastructure for notification and follow-up must be in place for Pap smear screening to provide improvements in outcome and has been shown to be different between racial groups [18]. Differences in follow-up for abnormal screening results may help explain later stage at the time of diagnosis, but they do not help explain the worse outcomes stage for stage amongst black women with cervical cancer.

Black women have been shown to be less likely to receive surgical therapy and more likely to receive radiotherapy when compared with white women [19]. Our study confirms this observation – we found that black patients were significantly less likely than whites to receive surgical treatment even when adjusted for stage at diagnosis and insurance status. These treatments constitute commonly prescribed standard and necessary treatments for cervical cancer.

Black women were also less likely to receive brachytherapy than white women. The administration of brachytherapy invariably requires access to a surgeon in order to place a brachytherapy device (tandem and ovoids, Smit sleeve, etc.). A treatment disparity for receipt of brachytherapy was previously described in 1998 by Mundt et al. In that study, black women were less likely to receive intracavitary radiation than white women for subjective reasons including patient refusal, medical comorbidities and technical problems. In comparison, the reason most commonly cited for white women to not receive brachytherapy following external beam radiation was objective – the presence of extra-pelvic disease [9]. These results demonstrate biases at the level of the physician, system and patient, are likely still present, and presumably account for some proportion of the disparity that we see in our study.

Although Maryland does have an adequate number of gynecologic oncologists, these specialists are distributed among a small number of specialized centers. Patients who live in large geographic swaths of the state may suffer a considerable travel burden to reach a gynecologic oncologist. In Prince George’s County, for example, cervical cancer incidence is 10–25% lower than the US rate, but cervical cancer mortality is 10–25% higher. This county has a large black population and no gynecologic oncology services.

This study is not without limitations. The dataset used for this study contained sufficient information to categorize the race of nearly all cases. However, as with all studies using state registries, our results should be read with an appreciation of the inherent limitations of cancer registries, including miscategorization of race (arising from self-reporting, observer reporting, insufficient granularity to distinguish “mixed race” individuals, etc.), reporting delays, the possibility of duplicate records, and the presumably very small number of cases that go unreported. Specifics of FIGO stage and surgical data were not available through this database. A percentage of variables were missing from our database, however analysis with and without missing variables yielded similar results. Lastly, this dataset does not capture survival data. Hence we are unable to know with absolute certainty whether our observed disparity in treatment accounts for observed statewide differences in disease specific mortality.

The State of Maryland serves as an excellent reflection of the United States population as a whole. The state has large populations of urban, suburban and rural constituents as well as a racial makeup that mirrors the United States. As the incidence of cervical cancer between white and black women in Maryland has become similar during the last decade, an opportunity exists to identify ways to reduce the inequalities in treatment that are potentially causative of a persistently higher mortality rate amongst black women in Maryland and in the United States.
